# Unilateral snow banking in tuberculosis-related intermediate uveitis

**DOI:** 10.1186/1869-5760-4-4

**Published:** 2014-02-10

**Authors:** Kalpana Babu, Soumya S Bhat

**Affiliations:** 1 Vittala International Institute of Ophthalmology, Bangalore 560085, India; 2 Prabha Eye Clinic & Research Centre, 504, 40th Cross, Jayanagar 8th Block, Bangalore 560070, India

**Keywords:** Intermediate uveitis, Snow banking, Tuberculosis, Antitubercular therapy

## Abstract

Snow banking is usually a term coined to describe the accumulation of vitreous exudates over the pars plana and the peripheral retina in pars planitis. Snow banking is very rare in tubercular intermediate uveitis. A 32-year-old male was diagnosed to have intermediate uveitis due to tubercular etiology in the right eye. Laboratory investigations include an increased erythrocyte sedimentation rate, positive Mantoux test, and computed tomography thorax showing mediastinal lymphadenopathy. Transbronchial needle aspiration of the lymph nodes showed chronic granulomatous inflammation with caseation. There were no recurrences following antitubercular therapy (ATT). This case report highlights the unique finding of snow banking in tubercular uveitis and course following treatment with ATT.

## Background

Snow banking is usually a term coined to describe the accumulation of white or cream vitreous exudates over the pars plana and the peripheral retina in pars planitis. It has also been described in intermediate uveitis due to Lyme disease, sarcoidosis, and multiple sclerosis [1-3]. Snow banking is very rare in tubercular intermediate uveitis. In this case report, we report an interesting finding of snow banking in tubercular intermediate uveitis and the response to antitubercular therapy (ATT).

## Case report

**Figure 1:**
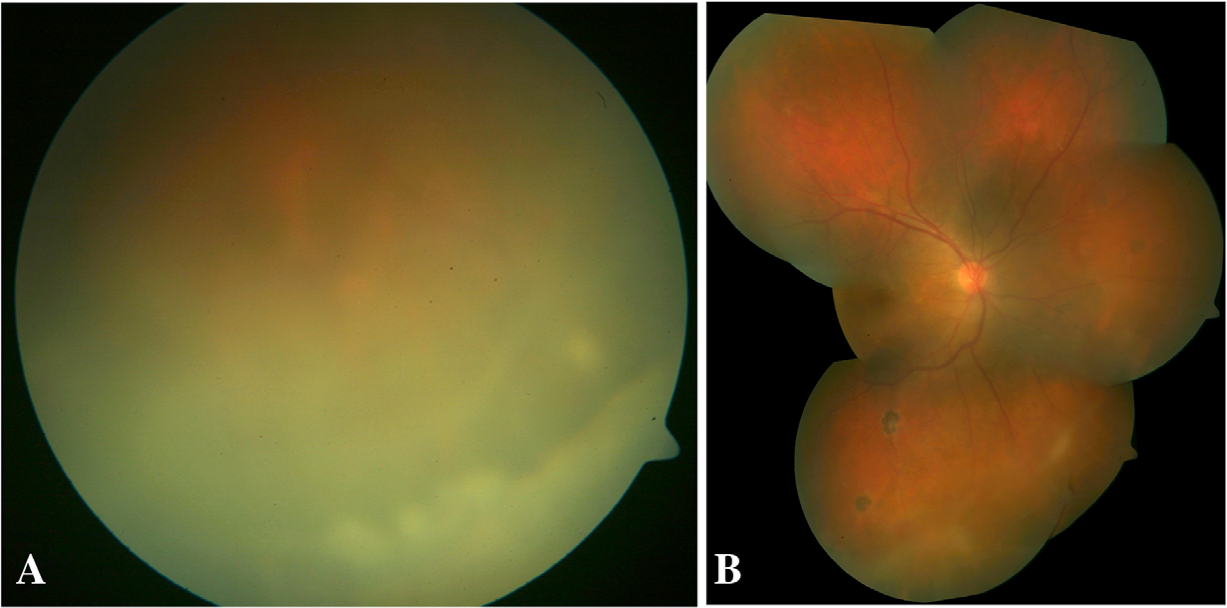
**Fundus photograph of the right eye.** Right eye showing inferior snow banking with vitreous exudates **(A)** with peripheral multifocal chorioretinal scar along the retinal blood vessel **(B)**.

**Figure 2:**
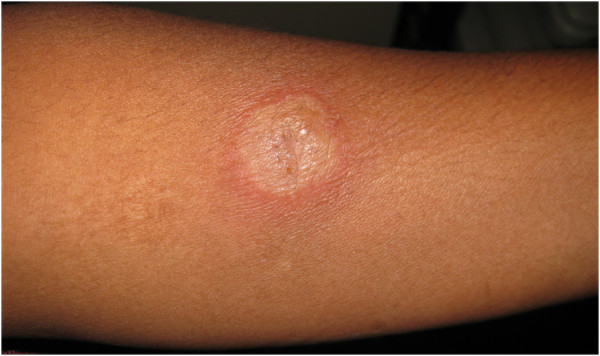
External photograph showing the positive Mantoux test.

**Figure 3:**
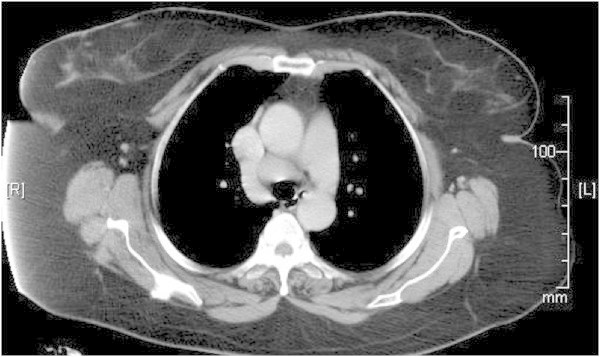
Computed tomography showing mediastinal lymphadenopathy.

**Figure 4:**
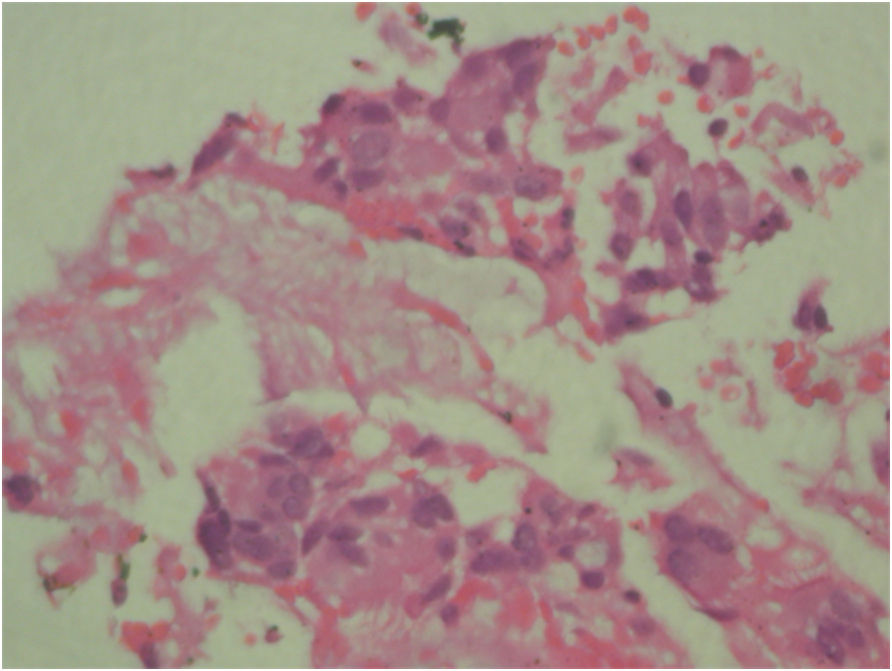
Transbronchial lymph node biopsy shows chronic granulomatous inflammation with caseation.

**Figure 5:**
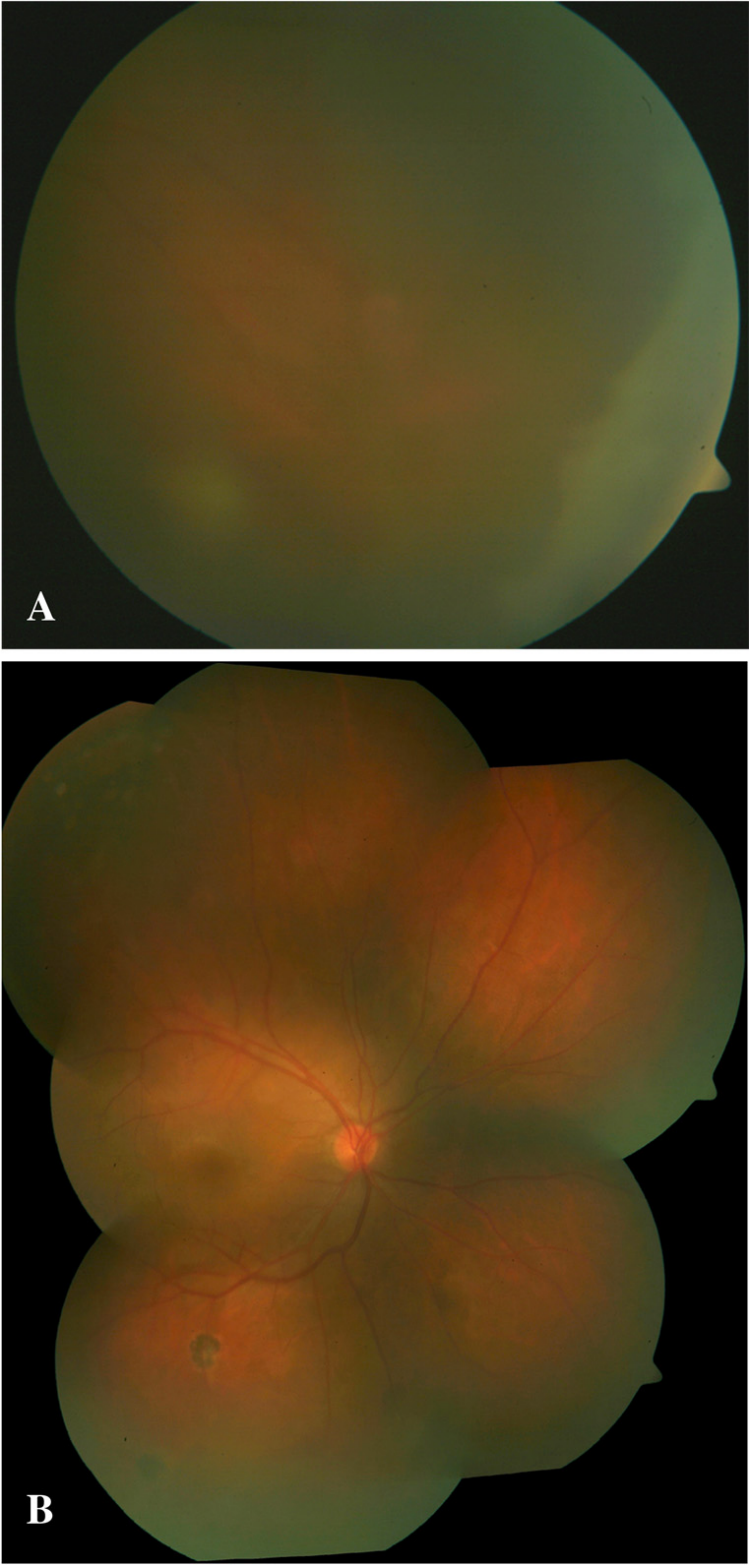
Fundus photograph of the right eye showing resolution of inflammation (A) and at last follow-up (B).

A 32-year-old male was referred to our institute for a history of diminution of vision and occasional redness in the right eye for a duration of 3 years. He was earlier treated with a course of oral steroids but had recurrences after stopping them. Systemic history was unremarkable. On examination, his best corrected visual acuity (BCVA) was 6/60 and 6/6 in OD and OS, respectively. Slit lamp examination showed anterior chamber (AC) cells 1+ (SUN working group classification [4]), round, reacting pupil, and vitreous haze of 1.5+ (SUN [4]) and vitritis. Hyperemia of the optic disc with cystoid macular edema, peripheral multifocal chorioretinal atrophic scars along retinal blood vessels, and snow banking in the pars plana and peripheral retina (Figure 1A,B) Left eye examination and intraocular pressures were normal. Laboratory investigations showed an increase in erythrocyte sedimentation rate (ESR) of 40 mm/h, positive Mantoux test (28-mm induration) (Figure 2), an increase in serum angiotensin converting enzyme, negative venereal disease research laboratory (VDRL) test, and normal liver function tests. Computed tomography (CT) of the thorax showed mediastinal lymphadenopathy with apical fibrosis in the lungs (Figure 3). Transbronchial needle aspiration (TBNA) of the lymph node showed chronic granulomatous inflammation with caseation (Figure 4). However, acid fast staining and culture for acid-fast bacilli were negative. ATT consisted of four drug regimens, which included isoniazid, rifampicin, pyrazinamide, and ethambutol in the first 2 months, isoniazid, rifampicin and pyrazinamide for the next 4 months, and isoniazid and rifampicin for the next 6 months. A single periocular injection of triamcinolone acetonide was given to the right eye along with oral steroids (40 mg/day in a tapering schedule over 8 weeks). Subsequent follow-ups showed improvement in vision to 6/9 (OD), resolving inflammation and scarring at the area of snow banking in the pars plana and peripheral retina. At 1.5-year follow-up following a 1-year complete course of ATT, his BCVA in the right eye was 6/6p. There was no anterior segment inflammation and no vitreous haze or vitritis. Scarring was seen in the peripheral retina and pars plana (Figure 5A,B). He has not had any recurrence of inflammation since then.

## Discussion

Intermediate uveitis is generally believed to be autoimmune in nature, and snow banking is an important clinical sign of intermediate uveitis [1-3]. In tuberculosis-endemic countries like India, tuberculosis is an important etiologic cause of intermediate uveitis [5]. Snow banking is generally described in intermediate uveitis due to autoimmune etiology and has not been described frequently in tubercular uveitis. This case report highlights the rare finding of snow banking in tubercular uveitis. It is important to investigate intermediate uveitis for tuberculosis even if snow banking is seen as specific antitubercular therapy helps in reducing the recurrences significantly.

## Abbreviations

ATT: antitubercular therapy; BCVA: best corrected visual acuity; CT: Computed tomography; TBNA: Transbronchial needle aspiration.

## Competing interests

The authors declare that they have no competing interests.

## Authors' contributions

KB was involved in the concept of the study, design, drafting of the manuscript, and revisions, while SSB was involved in the acquisition of data and drafting of the manuscript. Both authors read and approved the final manuscript.

## Authors' information

KB is a senior consultant in the Department of Uveitis and Intraocular Inflammation at Prabha Eye Clinic & Research Centre and Vittala International Institute of Ophthalmology, Bangalore. SSB is a junior fellow in the Department of Uveitis and Intraocular Inflammation at Prabha Eye Clinic & Research Centre and Vittala International Institute of Ophthalmology, Bangalore.
